# G Protein-Coupled Estrogen Receptor Immunoreactivity Fluctuates During the Estrous Cycle and Show Sex Differences in the Amygdala and Dorsal Hippocampus

**DOI:** 10.3389/fendo.2020.00537

**Published:** 2020-08-07

**Authors:** Ricardo Llorente, Marilena Marraudino, Beatriz Carrillo, Brigitta Bonaldo, Julia Simon-Areces, Pedro Abellanas-Pérez, Marina Rivero-Aguilar, Jose M. Fernandez-Garcia, Helena Pinos, Luis M. Garcia-Segura, Paloma Collado, Daniela Grassi

**Affiliations:** ^1^Department of Preclinical Odontology, Universidad Europea de Madrid, Madrid, Spain; ^2^Department of Neuroscience Rita Levi Montalcini, Neuroscience Institute Cavalieri Ottolenghi, University of Turin, Turin, Italy; ^3^Department of Psychobiology, Universidad Nacional de Educación a Distancia (UNED), Madrid, Spain; ^4^Instituto Mixto de Investigación Escuela Nacional de Sanidad-UNED (IMIENS), Madrid, Spain; ^5^Department of Physiotherapy, Podology and Dance, Universidad Europea de Madrid, Madrid, Spain; ^6^Cajal Institute, CSIC, Madrid, Spain; ^7^Centro de Investigación Biomédica en Red Fragilidad y Envejecimiento Saludable (CIBERFES), Instituto de Salud Carlos III, Madrid, Spain

**Keywords:** amygdala, hippocampus, estrous cycle, limbic system, GPER, estrogens, estrus, diestrus

## Abstract

G protein-coupled estrogen receptor (GPER) in the amygdala and the dorsal hippocampus mediates actions of estradiol on anxiety, social recognition and spatial memory. In addition, GPER participates in the estrogenic regulation of synaptic function in the amygdala and in the process of adult neurogenesis in the dentate gyrus. While the distribution of the canonical estrogen receptors α and β in the amygdala and dorsal hippocampus are well characterized, little is known about the regional distribution of GPER in these brain regions and whether this distribution is affected by sex or the stages of the estrous cycle. In this study we performed a morphometric analysis of GPER immunoreactivity in the posterodorsal medial, anteroventral medial, basolateral, basomedial and central subdivisions of the amygdala and in all the histological layers of CA1 and the dentate gyrus of the dorsal hippocampal formation. The number of GPER immunoreactive cells was estimated in these different structures. GPER immunoreactivity was detected in all the assessed subdivisions of the amygdaloid nucleus and dorsal hippocampal formation. The number of GPER immunoreactive cells was higher in males than in estrus females in the central (*P* = 0.001) and the posterodorsal medial amygdala (*P* < 0.05); higher in males than in diestrus females in the *strata orients* (*P* < 0.01) and *radiatum-lacunosum-moleculare* (*P* < 0.05) of CA1-CA3 and in the molecular layer of the dentate gyrus (*P* < 0.01); higher in diestrus females than in males in the basolateral amygdala (*P* < 0.05); higher in diestrus females than in estrus females in the central (*P* < 0.01), posterodorsal medial (*P* < 0.01) and basolateral amygdala (*P* < 0.01) and higher in estrus females than in diestrus females in the *strata oriens* (*P* < 0.05) and *radiatum-lacunosum-moleculare* (*P* < 0.05) of CA1-CA3 and in the molecular layer (*P* < 0.05) and the *hilus* of the dentate gyrus (*P* < 0.05). The findings suggest that estrogenic regulation of the amygdala and hippocampus through GPER may be different in males and in females and may fluctuate during the estrous cycle.

## Introduction

The hippocampus and the amygdala are two anatomically and functionally interconnected brain regions that participate in the regulation of stress responses (1, 2), fear (3–5), emotions (6–8), learning (9), and memory (8, 10). Both structures are integrated in the limbic system, which is altered in different pathological conditions, such as depression, anxiety, stress and schizophrenia, among others (11–20).

Some of the behaviors regulated by the hippocampus, the amygdala and their associated limbic structures are modulated by estradiol and testosterone (21–24) and are affected by sex (25–28) and by the phases of the estrous cycle (29–33). This hormonal regulation may be mediated by the modification of synaptic activity and plasticity in both the hippocampus (33–38) and the amygdala (29–31, 39, 40) and may represent a direct effect of testosterone and estradiol on these two brain structures, which express both androgen (41–43) and estrogen receptors (43–46).

Expression of classical estrogen receptors (ER)α and ERβ in the hippocampus and amygdala is well documented (44–47). After the discovery of the membrane-associated G protein-coupled estrogen receptor 1 (GPER), several studies have also explored its localization and function in the brain (48). GPER protein has been localized in the developing (49–51) and adult rodent hippocampus (52–59). In addition, GPER mRNA (60–62) and protein (63, 64) have been also detected in the adult rodent amygdala. However, the possible changes in GPER distribution in function of sex and the ovarian cycle in the hippocampus and amygdala have not been explored. Therefore, in this study we have analyzed the possible differences in GPER immunoreactivity between male, diestrus and estrus females in different anatomical subdivisions of the rat hippocampus and amygdala.

## Materials and Methods

### Animals and Experimental Procedure

Wistar albino male and female rats from our in-house colony were kept on a 12:12-h light–dark cycle and received food and water *ad libitum*. Animals were handled in accordance with the guidelines published in the “NIH Guide for the care and use of laboratory animals,” the principles presented in the “Guidelines for the Use of Animals in Neuroscience Research” by the Society for Neuroscience, and following the European Union (2010/63/UE) and the Spanish legislation (L6/2013; RD53/2013). Experimental procedures were approved by our Institutional Animal Use and Care Committee (UNED, Madrid). Special care was taken to minimize animal suffering and to reduce the number of animals used to the minimum necessary.

Twenty-four adult rats 2 months old (eight males and 16 females) were separately housed in plastic cages. After 2 weeks of habituation and handling, the monitoring of the estrous cycle in female rats was performed during 7 days by vaginal smears (65, 66). At the day 7, female rats were tested for the last vaginal smear in order to select the animals in estrus or diestrus (diestrus-2). Subsequently, all the animals, male and female, were deeply anesthetized with pentobarbital (Normon Veterinary Division, Madrid, Spain, 50 mg/kg) and perfused through the left cardiac ventricle with 50 ml of saline solution (0.9% NaCl) followed by 250 ml of fixative solution (4% paraformaldehyde in 0.1 M phosphate buffer, pH 7.4). Brains were quickly removed and immersed for 4–6 h at 4°C in the same fixative solution and then rinsed with phosphate buffer. Brains were placed for 72 h in a 30% sucrose solution in PBS, frozen in liquid isopentane at −35°C, and stored in a deep freezer at −80°C until sectioning. Brains were serially cut in the coronal plane at 20 μm thickness with a cryostat, obtaining 5 series of adjacent serial sections. In each series, each section was 100 μm distant from the following one. The plane of sectioning was oriented to match the drawings corresponding to the transverse sections of the rat brain atlas of Paxinos and Watson (67). Sections were collected in multiwell plates with a cryoprotectant solution and kept at −20°C. Immunohistochemical assay for GPER was performed on different series.

### Immunohistochemistry

The presence of GPER was detected by immunohistochemistry performed on free-floating sections according to the following steps. Before the reaction, the sections collected in the cryoprotectant solution were washed overnight at 4°C in PBS 0.1 M, pH 7.3–7.4. The following day, free floating sections were first washed for 30 min at room temperature in PBS 0.1 M, pH 7.3–7.4, containing 0.2% Triton X-100 and 0.2% BSA. Sections were then treated for 10 min with a solution of PBS 0.1 M, pH 7.3–7.4, containing methanol/hydrogen peroxide (PBS/methanol 1:1 with 0.3% hydrogen peroxide) to quench endogenous peroxidase activity. Sections were washed for 30 min at room temperature in PBS 0.1 M, pH 7.3–7.4, containing 0.2% Triton X-100 and 0.2% BSA and then incubated for 48 h at 4°C with a rabbit polyclonal GPER antibody (ABCAM, Cambridge, UK, reference ab39742) diluted 1:250 in 0.1 M PBS, pH 7.3–7.4, containing 0.2% Triton X-100, 0.2% BSA and 3% normal serum goat. A biotinylated goat anti-rabbit secondary antibody (Thermo scientific, Pierce, Rockford, IL, USA) was then used at a dilution of 1:300 for 120 min at room temperature. The antigen–antibody reaction was revealed by incubation with avidin-peroxidase complex (Thermo scientific, Pierce, Rockford, IL, USA) for 90 min. The peroxidase activity was visualized with a solution containing 0.187 mg/mL 3,3- diamino-benzidine (Sigma, Madrid, Spain) in PBS 0.1 M, pH 7.3–7.4. The sections were washed in the same buffer and collected on chromallum coated slides, air dried, cleared in xylene, and cover slipped with Depex (VWR International Eurolab, Barcelona, Spain) for quantitative analysis. One of each five consecutive sections was stained with 0.1% cresyl violet (pH 7.4) to facilitate the identification of the selected structures.

The GPER antibody used in the present study has been previously shown to recognize the full-length receptor protein in lysates of selected brain regions by Western blotting (68–72). Furthermore, immunostaining is abolished in rat brain sections when the GPER antibody is preincubated with the immunizing peptide (73). In agreement with our previous findings, GPER immunostaining was absent in rat brain sections preincubated with the GPER blocking peptide and when the first antibody was omitted.

### Morphometric Analysis

The morphometric analysis of GPER immunoreactive cells was performed on coded sections without knowledge of the experimental group. The number of GPER positive cells was assessed in the amygdala and the dorsal hippocampus using two coded sections per animal. Sections selected for analysis corresponded to the following coordinates: bregma −2.8 to −3.14 mm for the amygdaloid nucleus and bregma −2.8 to −3.8 mm for the dorsal hippocampal formation (67). The following regions were considered for the morphometric analysis of GPER immunoreactive cells: (i), the posterodorsal medial (MePD), anteroventral medial (MeAV), basolateral (BLA), basomedial (BMA), and central (CeM) amygdala; (ii), the *stratum oriens* (SO), the *stratum radiatum*, analyzed together with the *stratum lacunosum-moleculare* (SRLM) and the *stratum pyramidale* (SP) in dorsal Ammon's horn and (iii), the *stratum granulosum* (SG), the *stratum moleculare* (SM) and the *hilus* in the dorsal dentate gyrus.

Data presented for each region are the sum of the number of GPER immunolabeled cells in two brain sections per rat. For the amygdala, all cells located within the anatomical borders of each subnuclei were considered for quantification. Cresyl violet stained sections were used as reference for the delimitation of the analyzed structures. Given the anatomical heterogeneity of the hippocampus, counts were limited to the dorsal hippocampus and performed separately in CA1-CA3 and in the dentate gyrus. Cells were counted in eight fields from CA1-CA3, four fields from the *strata granulosum* and *moleculare* of the dentate gyrus and two fields from the *hilus*. Each field had an average area of 9.63 × 10^3^ μm^2^ for the SO; 7.49 × 10^3^ μm^2^ for the SP; 23.76 × 10^3^ μm^2^ for the SRLM; 6.52 × 10^3^ μm^2^ for the SG; 14.54 × 10^3^ μm^2^ for the SM and 21.5 × 10^3^ μm^2^ for the *hilus*. Selected fields were acquired by a digital camera (Olympus DP25) connected to a Nikon eclipse E600 microscope using x40 and x20 objectives. All GPER positive cells showing a cell nucleus and located within the boundary of the selected anatomical regions were included in the analysis, regardless of differences in cell shape, size and level of immunostaining. As a note of caution, it is important to consider that our morphometric approach is not unbiased from possible differences among the experimental groups in the volume of the anatomical structures analyzed. Thus, it should be considered a semi-quantitative estimation of the number of GPER positive cells.

### Statistical Analysis

Data were analyzed by one-way ANOVA followed by Bonferroni's *post-hoc* test, using the SPSS-17.0 software (SPSS Inc, Chicago, USA). A value of *P* < 0.05 was considered statistically significant. Data are presented as the mean ± SEM.

## Results

### Morphology of GPER Immunoreactive Cells

**Figure 1:**
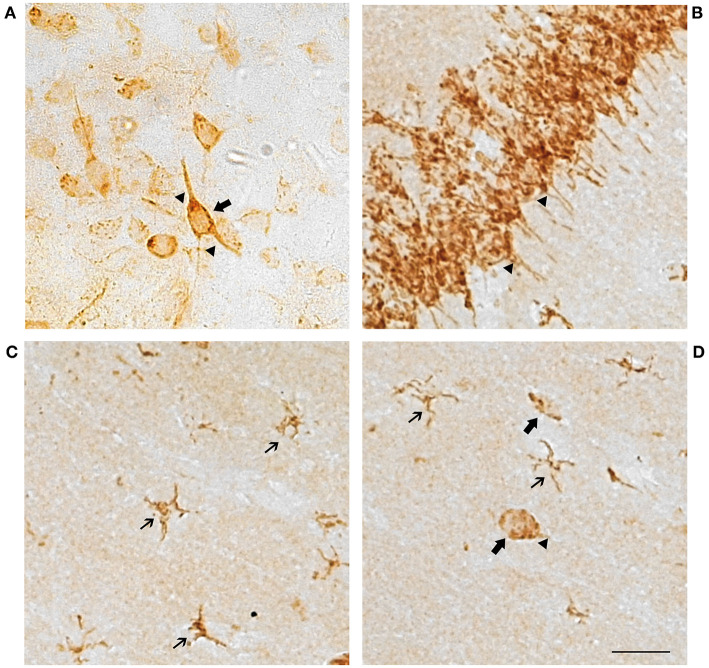
Representative GPER immunostaining showing cell perikaryon and the primary processes labeling in neuronal and glial cells. **(A)** Amygdaloid nucleus and **(B–D)** Hippocampal formation. Scale bar 10 μm. Thin arrows, GPER immunoreactive cells with glial morphology. Large arrows, GPER immunoreactive cells with neuronal morphology. Arrowheads, immunoreactive neuronal processes.

Cells showed a punctiform staining in the brain sections incubated with the GPER antibody (Figure 1). The staining was cytoplasmic, and the cell nucleus was always negative. Numerous cells showed a clear neuronal morphology with cytoplasmic immunostaining in the cell perikaryon and the primary dendritic processes. Dendritic staining was particularly evident in the pyramidal neurons of the hippocampus (Figure 1B), but it was also detected in neurons from the other studied regions (Figure 1A). In addition to neurons, a population of GPER immunoreactive cells showed a small perikaryon surrounded by tiny cell processes, a morphology that is characteristic of glial cells. These cells with glial morphology were observed in all the studied brain regions and in some of these regions, such as in the *stratum radiatum*, the *stratum lacunosum* and the *stratum moleculare* of the hippocampus, they represented the vast majority of the immunoreactive cells (Figures 1C,D).

### GPER Positive Cells in the Amygdaloid Nucleus

**Figure 2:**
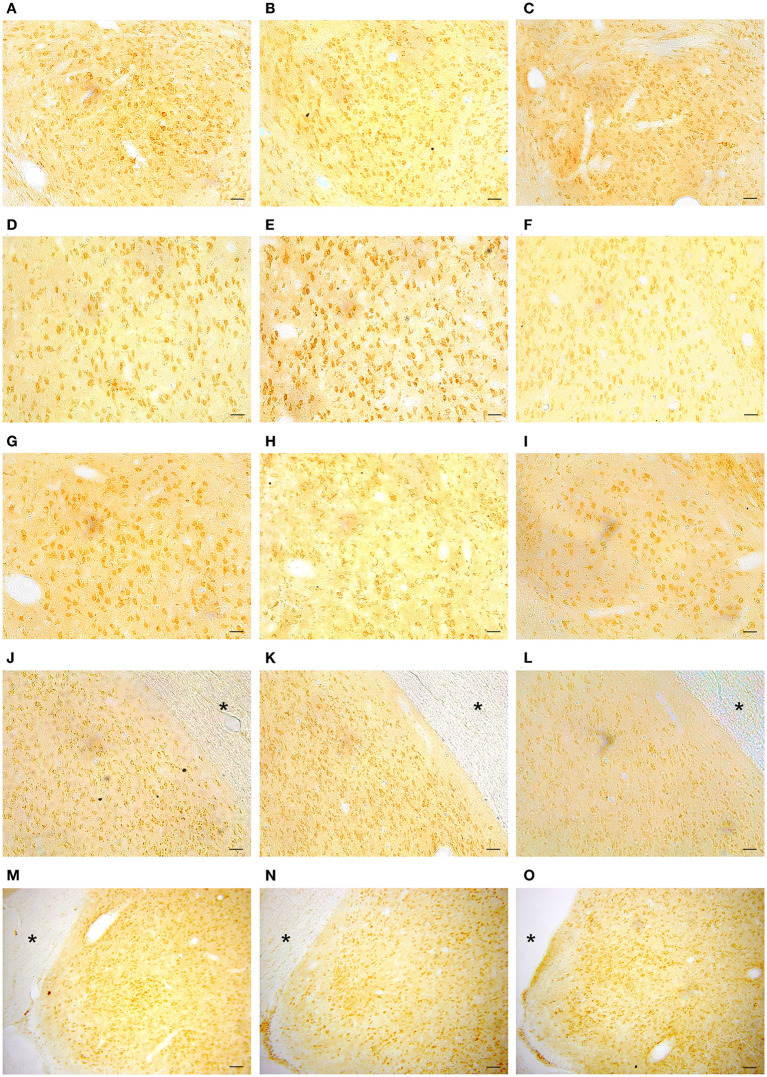
Representative examples of GPER immunohistochemical localization in rat amygdaloid nucleus in male animals (left column; **A,D,G,J,M**) and in females during diestrus (central column; **B,E,H,K,N**) and estrus (right column; **C,F,I,L,O**). **(A–C)** Central amygdala (CeM), **(D–F)** Basolateral amygdala (BLA), **(G–I)** Basomedial amygdala (BMA), **(J–L)** Medial posterodorsal amygdala (MePD), **(M–O)** Medial anteroventral amygdala (MeAV). *Optic tract. Scale bar, 50 μm.

**Figure 3:**
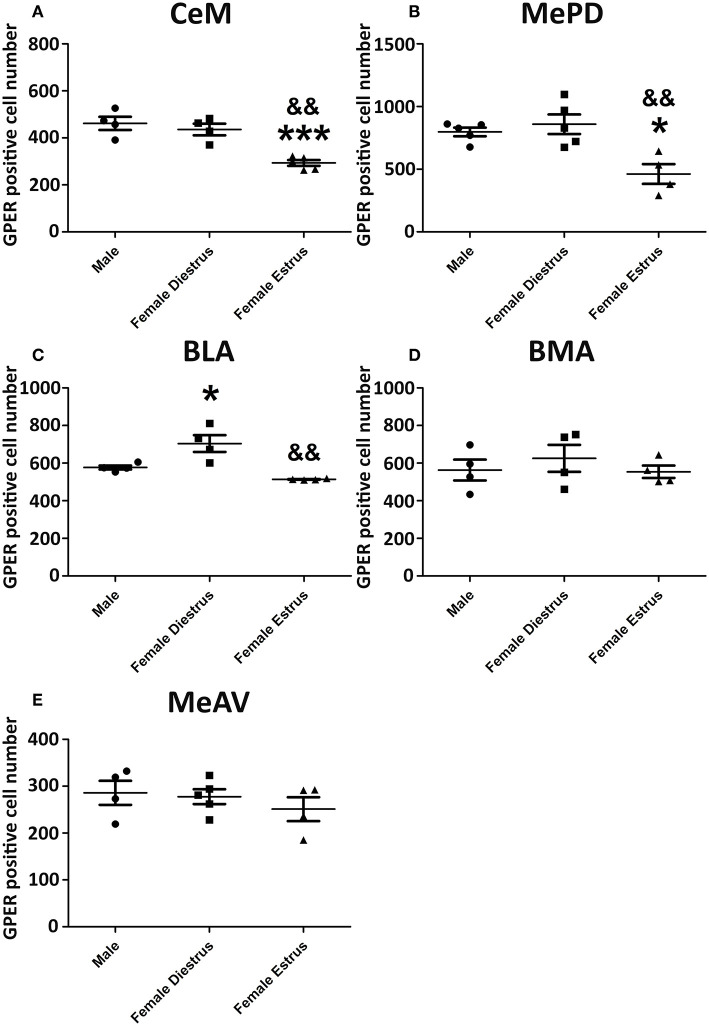
Number of GPER immunoreactive cells in the amygdaloid nucleus of male, diestrus females and estrus female rats. **(A)** Central amygdala. **(B)** Medial posterodorsal amygdala. **(C)** Basolateral amygdala. **(D)** Basomedial amygdala. **(E)** Medial anteroventral amygdala. Data are represented as mean±SEM. *, ***Significant differences (*p < 0.05 and ***p < 0.001) vs. male values. && Significant differences (*p* < 0.01) vs. females in diestrus.

Representative examples of GPER immunoreactivity in the amygdala of male and female animals are shown in Figure 2. Qualitative observation of GPER immunopositive cells in the amygdaloid nucleus revealed some differences in the pattern of staining among the different experimental groups. These differences were confirmed by the morphometric analysis. ANOVA analysis revealed significant differences among experimental groups in the central amygdala (CeM) [F_(2, 13)_ = 23.10; *P* = 0.001; Figure 3A], posterodorsal medial amygdala (MePD) [F_(2, 14)_ = 17.49; *P* = 0.002; Figure 3B] and basolateral medial amygdala (BLA) [F_(2, 12)_ = 25.89; *P* = 0.001; Figure 3C]. The *post-hoc* analysis revealed lower number of GPER immunopositive cells in estrus females that in males in the CeM (*P* = 0.001) and the MePD (*P* < 0.05) (Figures 3A,B). In contrast, females in diestrus showed a higher number of GPER immunoreactive cells than males in the BLA (*P* < 0.05) (Figure 2C). Moreover, estrus females showed a lower number of GPER immunoreactive cells than diestrus females in the CeM (*P* < 0.01), MePD (*P* < 0.01), and BLA (*P* < 0.01). No significant differences between the experimental groups were found in the basomedial (BMA) [F_(2, 12)_ = 0.828; *P* = 0.38; Figure 3D] and anteroventral medial (MeAV) amygdala [F_(2, 13)_ = 0.76; *P* = 0.41; Figure 3E].

### GPER Positive Cells in the Dorsal Hippocampus

**Figure 4:**
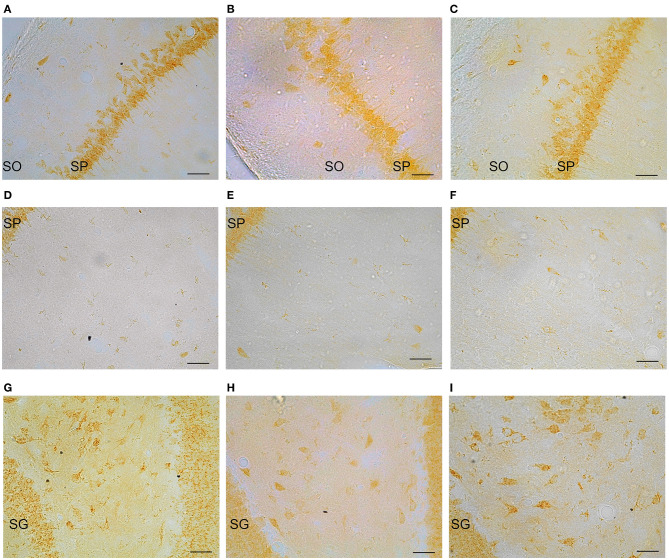
Representative examples of GPER immunohistochemical localization in rat hippocampal formation in male animals (left column; **A,D,G**) and in females during diestrus (central column; **B,E,H**) and estrus (right column; **C,F,I**). **(A–C)** *stratum oriens* (SO) and *stratum pyramidale* (SP) in CA1, **(D–F)** *stratum pyramidale* (SP) and *strata radiatum-lacunosum-moleculare* in CA1. **(G–I)** Dentate gyrus, *stratum granulare* (SG) and *hilus*. Scale bar, 20 μm.

**Figure 5:**
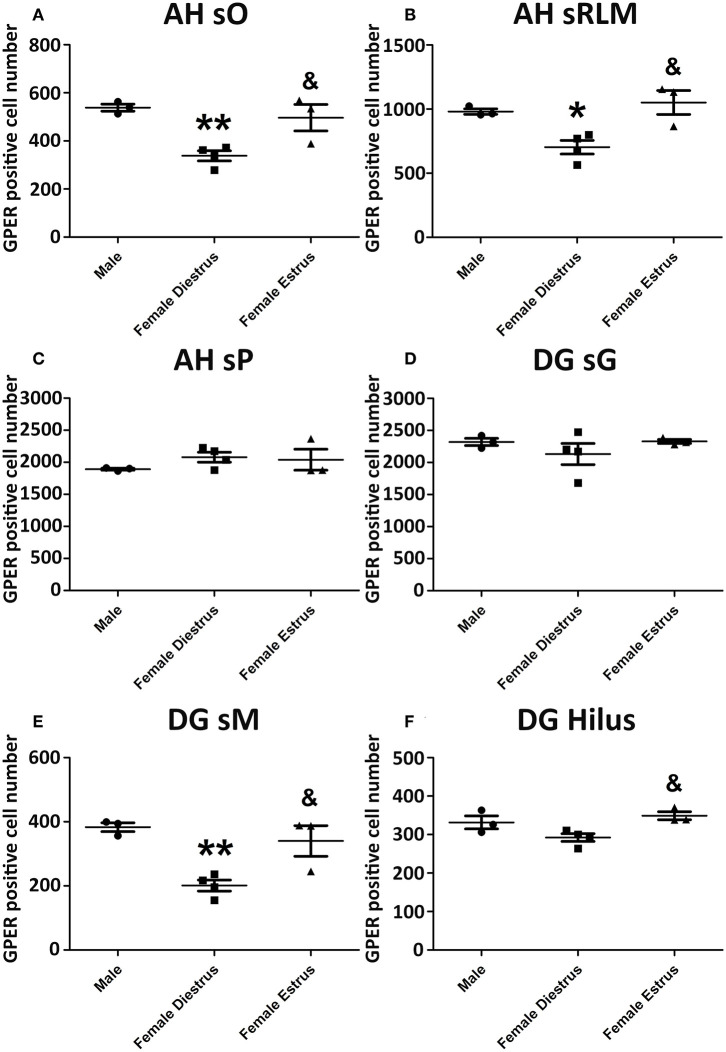
Number of GPER immunoreactive cells in the in hippocampal formation of male, diestrus females and estrus female rats. **(A)** Ammon's horn, *stratum oriens* (SO). **(B)** Ammon's horn, *strata radiatum-lacunosum-moleculare* (SRLM). **(C)** Ammon's horn, *stratum pyramidale* (SP). **(D)** Dentate gyrus, *stratum granulare* (SG). **(E)** Dentate gyrus, *stratum moleculare* (SM). **(F)** *Hilus*. Data are represented as mean±SEM. *, ** Significant differences (*p < 0.05; **p < 0.01) vs. male values. & Significant difference (*p* < 0.05) vs. females in diestrus.

Representative examples of GPER immunoreactive cells in the dorsal hippocampal formation are shown in Figure 4. ANOVA analysis showed significant differences in the *stratum oriens* (SO) [F_(2, 10)_ = 12.13; *P* = 0.01; Figure 5A] and the *strata radiatum-lacunosum-moleculare* (SRLM) [F_(2, 10)_ = 16.40; *P* = 0.005; Figure 5B]. The *post-hoc* analysis revealed a significantly lower number of GPER immunoreactive cells in diestrus females compared to males in the SO (*P* < 0.01) and the SRLM (*P* < 0.05). Moreover, diestrus females displayed also a lower number of GPER immunopositive cells than estrus female animals in the same regions: SO (*P* < 0.05) and SRLM (*P* < 0.05). In contrast, no significant differences among the experimental groups were detected in the *stratum pyramidale* (SP) [F_(2, 10)_ = 0.08; *P* = 0.78; Figure 5C].

Significant differences in the number of GPER immunoreactive cells among experimental groups were also detected in the dentate gyrus. Thus, ANOVA analysis showed significant differences in the *stratum moleculare* (SM) [F_(2, 10)_ = 12.69; *P* = 0.009; Figure 5E] and the *hilus* [F_(2, 10)_ = 10.89; *P* = 0.013; Figure 5F], but not in the *stratum granulare* (SG) [F_(2, 10)_ = 1.30; *P* = 0.29 Figure 5D]. Diestrus females showed a lower number of GPER immunoreactive cells than males in the SM (*P* < 0.01). In addition, diestrus females showed also a lower number of GPER immunopositive cells than estrus females in both the SM (*P* < 0.05) and the *hilus* (*P* < 0.05) (Figures 5E,F).

## Discussion

Previous studies have shown that GPER is widely distributed in the brain (50, 53, 55, 56, 60, 73). Indeed, GPER has been shown to be expressed by neurons, astrocytes and oligodendrocytes (56, 57, 59, 74–77) and GPER immunoreactivity has been detected by electron microscopy in both neuronal and glial profiles in the hippocampus (59), which is consistent with the detection of GPER immunoreactivity in cells with either neuronal or glial morphology in our study. Furthermore, we have detected a punctiform pattern of immunoreactivity that is absent in the cell nucleus, in agreement with the reported subcellular localization of GPER, either in the plasma membrane or in the endoplasmic reticulum and Golgi apparatus (52, 54, 57, 78–80).

To explore possible changes in GPER immunoreactivity during the estrous cycle we performed a semi-quantitative analysis of the number of GPER immunoreactive cells. Although our findings need to be confirmed by unbiased stereology, they suggest that the immunoreactive levels of GPER fluctuate during the estrous cycle in the amygdala and the dorsal hippocampus with regional specificity. Thus, significant differences in the number of GPER immunoreactive cells are observed between estrus and diestrus in the central, posterodorsal medial and basolateral amygdala; in the stratum oriens and the *strata radiatum-lacunosum-moleculare* of the Ammon's horn and in the molecular layer and the *hilus* of the dentate gyrus. These fluctuations in the number of GPER immunoreactive cells between estrous cycle stages are associated with transient sex differences in GPER immunoreactivity that are also regionally specific.

Our findings extend the results of previous studies showing changes during the estrous cycle in the number of GPER immunoreactive axonal, dendritic and glial profiles in the mouse hippocampal formation (59). Sex differences in GPER expression have been also reported in primary hippocampal neurons (49). Another study has reported increased GPER mRNA levels in the amygdala of male hamster compared to females (60). In addition, differences in the mRNA levels of GPER between different estrous cycle days have been detected in other rat brain regions, such as the nucleus of the solitary tract, the ventrolateral medulla and the periaqueductal gray (81).

One of the limitations of the immunohistochemical analysis is that it cannot discriminate between full length functional receptors and other inactive forms. Therefore, we can only speculate on the possible functional significance of the fluctuation in the number of GPER immunoreactive cells in the amygdala and hippocampus during the estrous cycle and the associated sex differences. Differences in GPER levels may contribute to synaptic changes during the estrous cycle in the posterodorsal medial amygdala, the basolateral amygdala, the central amygdala and Ammon's horn (82–86) and may be also associated with the fluctuation in adult neurogenesis in the dentate gyrus of adult females in response to the cyclic changes in plasma estradiol levels (33). Specifically, GPER has been shown to be involved in the regulation of excitatory and inhibitory transmission in the basolateral amygdala (61, 63, 86) and in the regulation of adult neurogeneis in the hippocampus (58). In addition, previous studies have shown that GPER in the basolateral amygdala mediates effects of estradiol on anxiety (64). Furthermore, GPER in the medial amygdala and the dorsal hippocampus participate in the modulation of social recognition by estradiol (23, 24, 87). Moreover, GPER in the dorsal hippocampus also mediates effects of estradiol on object recognition and spatial memory (23, 24, 87–90). Therefore, the observed modifications in GPER immunoreactivity in the amygdala and hippocampus may affect the actions of estradiol on these structures to regulate anxiety, social recognition, object recognition and spatial memory.

## Data Availability Statement

The raw data supporting the conclusions of this article will be made available by the authors, without undue reservation.

## Ethics Statement

The animal study was reviewed and approved by Universidad Nacional de Educación a Ditancia (UNED) bioethics comitee in compliance with the Spanish Royal Decree 53/2013 and the European Directive 2010/63/EU.

## Author Contributions

DG, LG-S, and PC designed and supervised the experiments. JF-G, RL, MM, DG, BB, BC, JS-A, PA-P, MR-A, and HP performed the experiments. RL and JS-A prepared the figures for publication. DG, RL, and LG-S wrote the first draft of the manuscript. All authors contributed to the article and approved the submitted version.

## Conflict of Interest

The authors declare that the research was conducted in the absence of any commercial or financial relationships that could be construed as a potential conflict of interest.
